# Insecticide-Treated Nets Can Reduce Malaria Transmission by Mosquitoes Which Feed Outdoors

**DOI:** 10.4269/ajtmh.2010.09-0579

**Published:** 2010-03

**Authors:** Nicodem J. Govella, Fredros O. Okumu, Gerry F. Killeen

**Affiliations:** Biomedical and Environmental Thematic Group, Coordination Office, Ifakara Health Institute, Dar es Salaam, Tanzania; Disease Control and Vector Biology Unit, London School of Hygiene and Tropical Medicine, London, United Kingdom; Vector Group, Liverpool School of Tropical Medicine, Liverpool, United Kingdom

## Abstract

Insecticide treated nets (ITNs) represent a powerful means for controlling malaria in Africa because the mosquito vectors feed primarily indoors at night. The proportion of human exposure that occurs indoors, when people are asleep and can conveniently use ITNs, is therefore very high. Recent evidence suggests behavioral changes by malaria mosquito populations to avoid contact with ITNs by feeding outdoors in the early evening. We adapt an established mathematical model of mosquito behavior and malaria transmission to illustrate how ITNs can achieve communal suppression of malaria transmission exposure, even where mosquito evade them and personal protection is modest. We also review recent reports from Tanzania to show that conventional mosquito behavior measures can underestimate the potential of ITNs because they ignore the importance of human movements.

Insecticide-treated nets (ITNs) represent a powerful means for controlling malaria in Africa.^1^ This usefulness is due to the fact that the principal malaria vectors, from the Giles *Anopheles gambiae* and *An. funestus* species complexes,^2^–^4^ primarily feed indoors at night.^2^,^5^,^6^ Thus, the proportion of human exposure that occurs indoors (π_i_), when persons are asleep and can conveniently use them, is very high (Figure 1A–D). Such estimates of π_i_, which take into consideration the movement patterns of persons are obtained in the field by weighting the observed indoor and outdoor biting rates at each period of the night by the proportion of humans that are typically in these two compartments at that time.^6^,^7^

**Figure 1.**
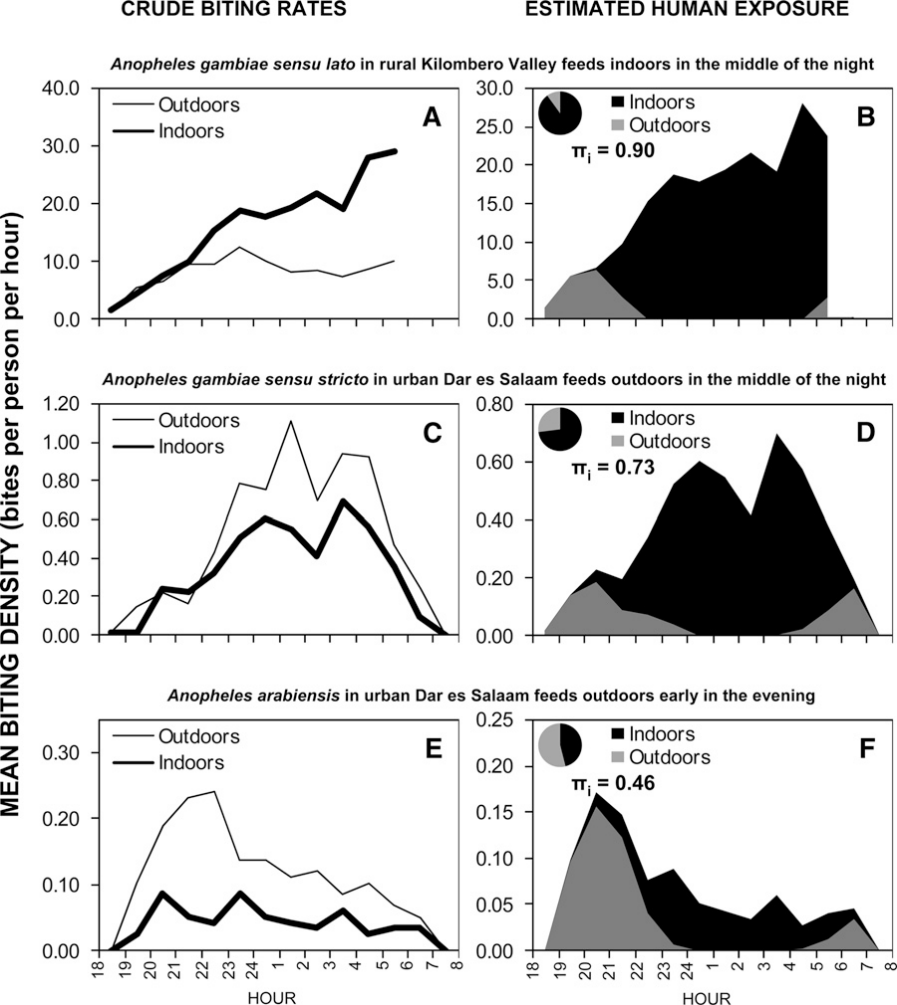
Crude behavioral profiles of three populations of malaria vectors in Tanzania (**A**, **C**, and **E**) and the corresponding exposure profiles of the human populations exposed to them (**B**, **D**, and **F**). The left panels plot crude behavioral profiles based on mean biting density of mosquitoes per hour, and the right panels represent human behavior-adjusted estimates of actual transmission exposure obtained by multiplying the mean biting density of mosquito in each hour and the proportion of humans present in the indoor and outdoor compartments.^6^,^7^

When reasonable levels of community-wide coverage are achieved, with approximately half of the population using them each night,^8^,^9^ ITNs not only confer personal protection against infectious bites but can also reduce the survival, feeding frequency, feeding success, and density of vector mosquito populations.^8^,^10^ This finding means that ITNs not only prevent malaria in protected persons, but can also reduce the exposure of unprotected persons by suppressing transmission across entire communities.^9^,^11^–^15^

Recent evidence suggests behavioral changes by malaria mosquito populations to avoid contact with ITNs by either feeding predominantly outdoors or in the early part of the evening.^5^,^7^,^16^–^18^ Such changes can drastically reduce the level of personal protection conferred by ITNs for obvious reasons.^5^,^7^ These behavioral changes might have resulted from the selection of genetically inherited traits or, more directly, from plastic phenotypic adaptation in response to increased coverage of ITNs or indoor residual spraying.^5^,^16^,^17^ Such intervention pressure may even be strong enough to cause changes in species composition of vector populations by selectively eliminating the most susceptible species and leaving those that are less vulnerable.^2^,^19^–^23^ For instance, *An. arabiensis* Patton, which is typically more exophilic, zoophagic, and exophagic than its sibling species *An. gambiae* sensu stricto, already dominates malaria transmission in parts of western Kenya where widespread use of ITNs has progressively diminished the importance of *An. gambiae* s.s as the main malaria vector.^20^

Although it is commonly perceived that ITNs are ineffective against outdoor-biting mosquitoes based on conventional measures of mosquito behavior,^5^,^18^,^24^ we adapt an established mathematical model of mosquito behavior and malaria transmission^8^,^10^ to examine the possibility that ITNs can achieve communal suppression of malaria transmission exposure, even where mosquito evade them and personal protection is modest. We adapt an existing model^8^ that was previously used to establish population-wide coverage thresholds levels of ITNs at which community-level protection is equivalent to or greater than personal protection.^8^ Specifically, we modify the model slightly to deal more realistically with vector populations that vary in terms of their feeding behaviors. The probability of mosquitoes surviving their eventual host attack (Pγ) is adjusted to account for the effect of ITN avoidance behavior, expressed as the proportion of normal exposure that would occur at times during which a human host would normally be under a net (π_i_). This parameter can also be thought of in simple terms as the maximum proportion of normal exposure, which is directly preventable through personal protection by using an ITN. The corrected probability of a mosquito surviving the eventual host attack is calculated with the following modification of equation 13 of the original model,^8^ assuming that the proportion of all attacks that end in death is the sum of mortality probabilities for attacking protected and unprotected hosts, weighted according to the proportion of the availability of all hosts that they represent.
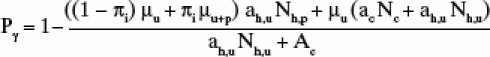


The definitions of relevant terms in the model are shown in Table 1. The reduction in relative rate of exposure (RRE) to malaria transmission achieved by individual-level personal protection (ITN users), community-level protection (ITN non-users), and combined individual and communal protection (ITN users) was estimated by fixing the additional mortality probability of mosquitoes encountering an ITN at 0.8^25^ and ITN coverage at the achievable level of 0.5, equivalent to 50% use as recorded in typical household surveys and specified by internationally agreed targets.^8^,^9^ Otherwise, the model is formulated, parameterized, and applied exactly as previously described.^8^

Figure 1E and F show that less than half of all human exposure to *An. arabiensis* in urban Dar es Salaam, Tanzania^7^ occurs in times and places when using an ITNs is feasible (π_i_ = 0.46). Based on these published field data, simulations predict only a slight suppression in personal relative rate of exposure to transmission (RRE = 0.59), equivalent to a 1.7-fold reduction (Figure 2). However, much greater decreases in exposure to transmission for ITN users (communal plus personal protection; RRE = 0.19) and non users (communal protection only; RRE = 0.32) are predicted at 50% community-wide coverage. Thus, even non-users receiving only communal protection can expect 3.1-fold reduction of exposure to transmission and users enjoy a 5.3-fold reduction. Extrapolating this level of communal protection horizontally across Figure 2 shows that this is equivalent to the personal protection provided when mosquitoes feed predominantly at times when most resident are indoors (π_i_ = 0.77). Once reasonably high use rates are attained, communal protection achieved is greater than personal protection because even modest reductions of mosquito survival and feeding success per gonotrophic cycle result in much larger impacts upon proportion of mosquitoes surviving the multiple blood feeds required to reach an age where they can transmit mature sporogonic-stage parasites.^26^–^28^

**Figure 2.**
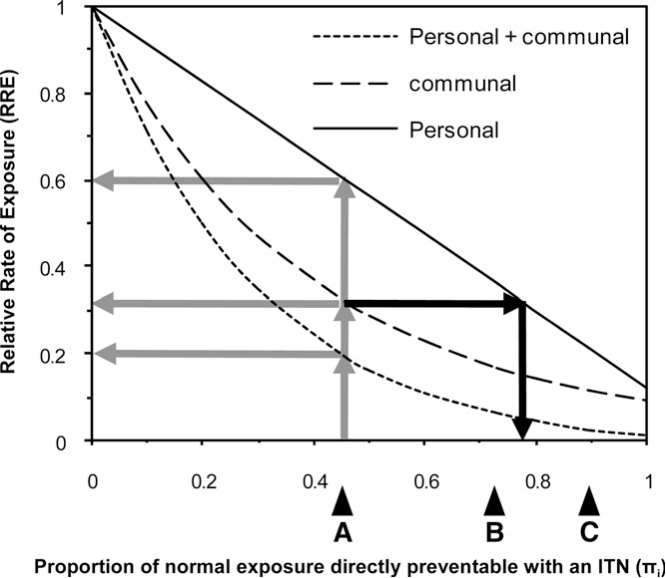
Simulated relationship between personal (users), communal (non-users), and combined effect of personal and communal (users) level suppression of malaria transmission exposure across a range values for the proportion of normal exposure for an unprotected individual occurring at times when insecticide-treated nets (ITNs) would be in use if they were available (π_i_). **Arrows A**, **B**, and **C** represent reported values of π_i_ for *Anopheles arabiensis* and *An. gambiae* s.s in urban Dar es Salaam, Tanzania^7^ and *An. gambiae* sensu lato in the rural Kilombero Valley,^6^ respectively (Figure 1). Note that although here we present a scenario in which overall ITN coverage level is set at 50%, the degree of personal protection against exposure is independent of coverage in the community at large.

Conventional mosquito behavior measures^5^,^18^,^24^,^29^–^32^ can underestimate the potential of ITNs because they ignore the importance of human movements^33^ indoors and outdoors. *Anopheles gambiae* s.s*.* also prefers to bite outdoors in Dar es Salaam (Figure 1C),^7^ but surveys of human malaria prevalence confirm that ITNs confer valuable personal protection and reduce infection risk by 23.6% (95% confidence interval = 61.4–95.1%, *P* = 0.016.^34^ This finding is due to the fact that because persons sleep indoors during peaks of mosquito activity, this location is where most human exposure occurs (π_i_ = 0.73; Figure 1D), and can be prevented by using an ITN.^7^

Plotting π_i_ versus the proportion of mosquitoes that are caught indoors by conventional field methods (Figure 3) shows that in all cases, the latter consistently underestimates the former. Even for highly exophagic populations of mosquitoes, most bites (Figure 3) can be confined to times when most humans are indoors^7^ and possibly under a net. This approach can therefore underestimate the full potential of ITNs because it considers outdoor catches at times when they have little or no epidemiologic relevance. Conversely, the proportion of mosquitoes that are caught at times during which most persons are asleep can overestimate or underestimate π_i_ for exophagic and endophagic vectors, respectively, because outdoor catches during these period and indoor catches in the evenings and mornings are included (Figure 3).

**Figure 3.**
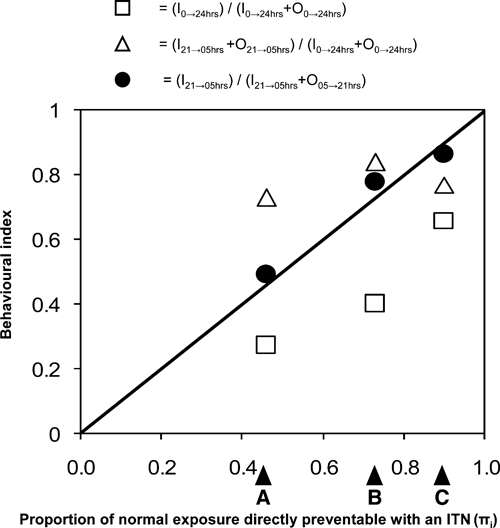
Graph of three crude behavioral indices for three populations of *Anopheles* mosquitoes in Tanzania compared with formal estimates of π_i_, which is the maximum proportion of normal exposure that is directly preventable by using an insecticide-treated net. **Arrows A**, **B**, and **C** represent reported values of π_i_ for *An. arabiensis* and *An. gambiae* s.s in urban Dar es Salaam^7^ and *An. gambiae* sensu lato in the rural Kilombero Valley,^6^ respectively (Figure 1). Open squares represent the proportion of mosquitoes that are caught indoors calculated by dividing the total catch indoors across all times (I_0→24 hours_) by total catch occurring outdoors (O_0→24 hours_) and indoors (I_0→24 hours_). The open triangles represent the proportion of mosquitoes that are caught at times when most humans are likely asleep, obtained by dividing the total catch occurring indoors and outdoors from 9:00 pm to 5:00 am (I_9:00 pm→5:00 am_ + O_9:00 pm→5:00 am_) by total catch indoors and outdoors across all times (I_0→24 hours_ + O_0→24 hours_). The filled circles represents a crude estimate of the proportion of exposure occurring indoor (π_i_), obtained by dividing the total catch occurring indoor from 9:00 pm to 5:00 am hours (I_9:00 pm → 5:00 am_) by itself plus the total outdoor catch from 5:00 am hours to 9:00 pm (O_5:00 am→9:00 pm_).

However, the number of mosquitoes caught indoors during sleeping hours, expressed as a proportion of itself plus the number mosquitoes caught outdoors outside of sleeping hours, closely matches formal estimates of π_i_ (Figure 3). Although the level of exophagy and endophagy of vector populations influences the efficacy of ITNs for preventing malaria transmission, human movement patterns and the extent to which vector activity patterns match them may often be more important. These examples from Dar es Salaam^7^ illustrates how two exophagic vector populations can avoid ITNs to different extents because of differences in their peak times of activity and the degree to which these coincide with human behavioral patterns. In simple terms, it is more important that persons are asleep and can conveniently use an ITN when vector activity peaks than that the place they sleep is preferred by those mosquitoes.

We therefore caution that ITNs should not be automatically discarded as a priority vector control measure just because vector mosquitoes are observed to prefer feeding outdoors. Explicit estimates of π_i_ values for locally relevant populations should first be obtained in the field and the potential community-level benefits, which are rarely captured by standard survey designs, should be carefully considered. Personal protection measures such as spatial repellents^35^,^36^ may be required to protect against outdoor bites in the morning or early evening,^16^,^29^,^37^ but should only be considered a supplement to ITNs unless proven otherwise. If the equitable, population-wide benefits of communal protection are ignored, potential opportunities for effective malaria control with a well-proven existing technology may be missed because the requirements for behaviorally-susceptible vector populations may be overestimated or overemphasized.

## Figures and Tables

**Table 1:** Parameter definitions

P_γ_ = Mean probabilities of surviving eventual host attack
π_i_ = Proportion of normal exposure of unprotected humans lacking nets that occurs at times and places when net users would be protected by sleeping under them
μ_u_ = Mortality upon attacking an unprotected host
μ_u+p_ = Overall mortality upon attacking a protected host
a_h,u_ = Mean availability of individual unprotected humans
N_h,p_ = Number of protected humans
a_c_ = Mean availability of individual cattle
N_c_ = Number of cattle
N_h,u_ = Number of unprotected humans
N_h_ = Number of humans
A_c_ = Total availability of cattle

## References

[1] Lengeler C 2004 Insecticide-treated bed nets and curtains for preventing malaria Cochrane Database Syst Rev CD000363 15106149 10.1002/14651858.CD000363.pub2

[2] Gillies MT DeMeillon B 1968 The Anophelinae of Africa South of the Sahara (Ethiopian Zoogeographical Region) Johannesburg South African Institute for Medical Research

[3] Gillies MT Coetzee M 1987 A Supplement to the Anophelinae of Africa South of the Sahara (Afrotropical Region) Johannesburg South African Medical Research Institute

[4] White GB 1974 *Anopheles gambiae* complex and disease transmission in Africa Trans R Soc Trop Med Hyg 68 279 301 10.1016/0035-9203(74)90035-2 4420769

[5] Pates H Curtis C 2005 Mosquito behavior and vector control Annu Rev Entomol 50 53 70 15355233 10.1146/annurev.ento.50.071803.130439

[6] Killeen GF Kihonda J Lyimo E Oketch FR Kotas ME Mathenge E Schellenberg JA Lengeler C Smith TA Drakeley CJ 2006 Quantifying behavioural interactions between humans and mosquitoes: evaluating the protective efficacy of insecticidal nets against malaria transmission in rural Tanzania BMC Infect Dis 6 161 17096840 10.1186/1471-2334-6-161 PMC1657018

[7] Geissbühler Y Chaki P Emidi B Govella NJ Shirima R Mayagaya V Mtasiwa D Mshinda H Fillinger U Lindsay SW Kannady K Caldas de Castro M Tanner M Killeen GF 2007 Interdependence of domestic malaria prevention measures and mosquito-human interactions in urban Dar es Salaam, Tanzania Malar J 6 126 17880679 10.1186/1475-2875-6-126 PMC2039744

[8] Killeen GF Smith TA Ferguson HM Mshinda H Abdulla S Lengeler C Kachur SP 2007 Preventing childhood malaria in Africa by protecting adults from mosquitoes with insecticide-treated nets PLoS Med 4 e229 17608562 10.1371/journal.pmed.0040229 PMC1904465

[9] Hawley WA Phillips-Howard PA Terkuile FO Terlouw DJ Kolczak MS Hightower AW 2003 Community-wide effects of permethrin-treated bed nets on child mortality and malaria morbidity in western Kenya Am J Trop Med Hyg 68 Suppl 4 121 127 12749495

[10] Killeen GF Smith TA 2007 Exploring the contributions of bed nets, cattle, insecticides and excitorepellency to malaria control: a deterministic model of mosquito host-seeking behaviour and mortality Trans R Soc Trop Med Hyg 101 867 880 17631372 10.1016/j.trstmh.2007.04.022 PMC1949412

[11] Gimnig JE Kolczak MS Hightower AW Vulule JM Schoute E Kamau L Phillips-Howard PA ter Kuile FO Nahlen BL Hawley WA 2003 Effect of permethrin-treated bed nets on the spatial distribution of malaria vectors in western Kenya Am J Trop Med Hyg 68 115 120 12749494

[12] Maxwell CA Msuya E Sudi M Njunwa KJ Carneiro IA Curtis CF 2002 Effect of community-wide use of insecticide-treated nets for 3–4 years on malarial morbidity in Tanzania Trop Med Int Health 7 1003 1008 12460390 10.1046/j.1365-3156.2002.00966.x

[13] Hii JLK Smith T Vounatsou P Alexander N Mai A Ibam E Alpers MP 2001 Area effects of bednet use in a malaria-endemic area in Papua New Guinea Trans R Soc Trop Med Hyg 95 7 13 11280071 10.1016/s0035-9203(01)90315-3

[14] Binka FN Indome F Smith T 1998 Impact of spatial distribution of permethrin-impregnated bed nets on child mortality in rural northern Ghana Am J Trop Med Hyg 59 80 85 9684633 10.4269/ajtmh.1998.59.80

[15] Howard SC Omumbo J Nevill CG Some ES Donnelly CA Snow RW 2000 Evidence for a mass community effect of insecticide treated bednets on the incidence of malaria on the Kenyan coast Trans R Soc Trop Med Hyg 94 357 360 11127232 10.1016/s0035-9203(00)90103-2

[16] Braimah N Drakeley C Kweka E Mosha FW Helinski M Pates H Maxwell CA Massawe T Kenward MG Curtis C 2005 Tests of bednet traps (Mbita traps) for monitoring mosquito populations and time of biting in Tanzania and possible impact of prolonged ITN use Int J Trop Insect Sci 25 208 213

[17] Charlwood JD Graves PM 1987 The effect of permethrin-impregnated bednets on a population of *Anopheles farauti* in coastal Papua New Guinea Med Vet Entomol 1 319 327 2979548 10.1111/j.1365-2915.1987.tb00361.x

[18] Oyewole IO Awolola TS 2006 Impact of urbanization on bionomics and distribution of malaria vectors in Lagos, southwestern Nigeria J Vector Borne Dis 43 173 178 17175702

[19] Gillies MT Smith A 1960 Effect of a residual house spraying campaign on species balance in *Anopheles funestus* group: the replacement of *Anopheles funestus* Giles with *Anopheles rivulorum* Leeson Bull Entomol Res 51 248 252

[20] Lindblade KA Gimnig JE Kamau L Hawley WA Odhiambo F Olang G Terkuile FO Vulule JM Slutsker L 2006 Impact of sustained use of insecticide-treated bednets on malaria vector species distribution and Culicine mosquitoes J Med Entomol 42 428 432 16619629 10.1603/0022-2585(2006)043[0428:iosuoi]2.0.co;2

[21] Odetoyinbo JAA Davidson G 1968 The Anopheles gambiae Complex and its Role in Malaria Transmission in the Islands of Zanzibar and Pemba, United Republic of Tanzania WHO/MAL 68 Geneva World Health Organization

[22] Gillies MT Furlong M 1964 An investigation into behaviour of *Anopheles parensis* Gillies at Malindi on coast of Kenya Bull Entomol Res 55 1 16

[23] Gillies MT 1962 A new species of the *Anopheles funestus* complex (Diptera: Culicidae) from East Africa Proc R Entomol Soc London (B) 31 81 86

[24] Rubio-Palis Y Curtis CF 1992 Biting and resting behaviour of Anophelines in western Venezuela and implications for control of malaria transmission Med Vet Entomol 6 325 334 1463897 10.1111/j.1365-2915.1992.tb00628.x

[25] Graham K Kayedi MH Maxwell C Kaur H Rehman H Malima R Curtis CF Lines JD Rowland MW 2005 Multicountry field trials comparing wash-resistance of PermaNet and conventional insecticide-treated nets against anopheline and culicine mosquitoes Med Vet Entomol 19 72 83 15752180 10.1111/j.0269-283X.2005.00543.x

[26] Garrett-Jones C 1964 Prognosis for interruption of malaria transmission through assessment of the mosquito's vectorial capacity Nature 204 1173 1175 14268587 10.1038/2041173a0

[27] Garrett-Jones C Shidrawi GR 1969 Malaria vectorial capacity of a population of *Anopheles gambiae* Bull World Health Organ 40 531 545 5306719 PMC2556109

[28] MacDonald G 1957 The Epidemiology and Control of Malaria London Oxford University Press

[29] Sungvornyothin S Muenvorn V Garros C Manguin S Prabaripai A Bangs MJ Chareonviriyaphap T 2006 Trophic behavior and biting activity of the two sibling species of the *Anopheles minimus* complex in western Thailand J Vector Ecol 31 252 261 17249342 10.3376/1081-1710(2006)31[252:tbabao]2.0.co;2

[30] Krafsur ES 1971 Malaria transmission in Gambela, Illubabor province Ethiop Med J 9 75 5149953

[31] White GB 1973 Comperative studies on sibling species of the *Anopheles gambiae* Giles complex (Dipt. Culicidae). III. The distribution, ecology, behaviour and vectorial importance of species D in Bwamba County, Uganda, with an analysis of biological, ecological, morphological and cytogenetical relationship of Ugandan species D Bull Entomol Res 63 65 97

[32] Henry M Gelfand MD 1955 *Anopheles gambiae* Giles and *Anopheles melas* Theobald in a coastal area of Liberia, West Africa Trans R Soc Trop Med Hyg 49 508 527 13281920 10.1016/0035-9203(55)90023-7

[33] Stoddard ST Morrison AC Vazquez-Prokopec GM Pazsoldan V Kochel TJ Kitron U Elder JP Scott TW 2009 The role of human movement in the transmission of vector-borne pathogen PLoS Negl Trop Dis 3 e481 19621090 10.1371/journal.pntd.0000481 PMC2710008

[34] Geissbühler Y Kannady K Chaki PP Emidi B Govella NJ Mayagaya V Kiama M Mtasiwa D Mshinda H Lindsay SW 2009 Microbial larvicide application by a large-scale, community-based program reduces malaria infection prevalence in Urban Dar Es Salaam, Tanzania PLoS One 4 e5107 19333402 10.1371/journal.pone.0005107 PMC2661378

[35] Pates HV Line JD Keto AJ Miller JE 2002 Personal protection against mosquitoes in Dar es Salaam, Tanzania, by using a kerosene oil lamp to vaporize transfluthrin Med Vet Entomol 16 277 284 12243228 10.1046/j.1365-2915.2002.00375.x

[36] Seyoum A Killeen GF Kabiru EW Knols BG Hassanali A 2003 Field efficacy of thermally expelled or live potted repellent plants against African malaria vectors in western Kenya Trop Med Int Health 8 1005 1011 14629767 10.1046/j.1360-2276.2003.01125.x

[37] Trung HD Bortel WV Sochantha T Keokenchanh K Briet OJ 2005 Behavioural heterogeneity of *Anopheles* species in ecologically different localities in southeast Asia: a challenge for vector control Trop Med Int Health 10 251 262 15730510 10.1111/j.1365-3156.2004.01378.x

